# Clinical Validation of Object Detection Models for AI-Based Pressure Injury Stage Classification

**DOI:** 10.3390/diagnostics16050747

**Published:** 2026-03-02

**Authors:** Sang Hyun Jang, Chunhwa Ihm, Jun-Woo Choi, Dong-Hun Han, Kyunghwa Bae, Minsoo Kang

**Affiliations:** 1 Department of Neurology, Daejeon Medical Center, Eulji University, Daejeon 35233, Republic of Korea; mj@eulji.ac.kr; 2 Department of Laboratory Medicine, Daejeon Medical Center, Eulji University, Daejeon 35233, Republic of Korea; haneul@eulji.ac.kr; 3 Department of Medical Artificial Intelligence, Eulji University, Seongnam 13135, Republic of Korea; chlwnsdn456@naver.com (J.-W.C.); d555v@naver.com (D.-H.H.); 4 Department of Quality improvement, Daejeon Medical Center, Eulji University, Daejeon 35233, Republic of Korea; sori1004@eulji.ac.kr; 5 Department of Bigdata Medical Convergence, Eulji University, Seongnam 13135, Republic of Korea

**Keywords:** pressure injury, artificial intelligence, object detection, image processing, deep learning, YOLO, medical applications

## Abstract

**Background/Objectives**: Pressure injury stage classification was performed using object detection models to address inconsistencies in clinical assessment due to variability in nurses’ experience and education levels. **Methods**: A dataset of 1282 pressure injury images from a medical institution was used to train and compare five representative architectures, YOLOv5x, YOLOv7, YOLOv8x, YOLOv8n, and YOLOv11x, and Faster R-CNN across Stages 1–4, excluding Deep Tissue Injury and unclassified cases. A mobile application incorporating YOLOv7 was deployed at Eulji University Daejeon Medical Center and tested by 10 nurses over 2 weeks, processing 46 cases. **Results**: YOLOv7 demonstrated superior performance with mAP@0.5 of 0.97 and mAP@0.5:0.95 of 0.68, achieving 93% accuracy for Stage 2 classification, the most challenging diagnostic category. Clinical validation demonstrated 87% diagnostic accuracy, 4.0/5 user satisfaction, and workflow improvement with assessment time reduced from 4–6 min to 1 min. The application proved valuable as both a diagnostic support tool and educational resource for novice nurses, with zero critical misclassifications recorded. **Conclusions**: This study establishes the practical utility of AI-based pressure injury classification systems in clinical practice and their potential for enhancing nursing competency and workflow efficiency.

## 1. Introduction

Pressure ulcers represent a significant clinical challenge, typically resulting from sustained mechanical forces that compromise vascular supply, predominantly affecting individuals with restricted mobility or sensorimotor deficits [1]. Clinical staging follows the NPUAP/EPUAP framework, categorizing lesions into Stages 1 through 4, along with Unstageable and Deep Tissue Injury (DTI) classifications [2]. Accurate staging is critical for determining appropriate treatment protocols; however, visual assessment is often subjective and inconsistent, compounded by persistent educational deficiencies among nursing staff globally [3,4,5,6,7,8,9].

This situation necessitates supplementary support mechanisms for inadequately trained personnel. With the increasing adoption of mobile technologies in healthcare, Artificial Intelligence (AI) has emerged as a promising solution to standardize assessment and support decision-making [10]. Mobile application-based approaches have previously demonstrated feasibility for wound assessment and management in clinical settings [11], providing a translational foundation for AI-assisted staging tools. While previous studies have explored AI for wound classification, most have focused on model performance metrics in experimental settings without validating clinical integration into actual nursing workflows, and none have prospectively assessed usability and workflow impact in a real ICU deployment. Furthermore, existing studies have been limited by small datasets, inconsistent class definitions, and the absence of clinical feasibility evidence. This study addresses these gaps through the following objectives: (1) to systematically compare multiple object detection architectures for automated pressure ulcer staging under standardized transfer learning conditions; (2) to identify the best-performing architecture for potential clinical deployment; (3) to prospectively evaluate the feasibility, usability, and workflow impact of the best-performing model through real-world deployment in an ICU nursing workflow.

### Related Works

Recent computational approaches to pressure ulcer management have demonstrated significant potential across multiple methodological paradigms. A 2021 study leveraged electronic health records (EHR) and administrative datasets to establish predictive models for pressure ulcer development risk [12]. The research identified prolonged bed rest, sensory impairment, postural maintenance difficulties, and hospitalization duration as primary contributing factors. Employing Random Forest methodology, researcher archived an AUC of 0.864, substantially exceeding conventional clinical assessment tools. This classification capability differential underscores the capacity of AI frameworks to enhance preventive strategies and early intervention protocols within clinical environments.

Traditional risk assessment instruments, while clinically validated, frequently exhibit constrained predictive capabilities and may impose additional administrative burden on nursing personnel. Addressing these limitations, researchers proposed an EHR-based daily risk prediction framework [13]. The study utilized approximately 30,000 textual records spanning one year from general medical units within a major Dutch medical facility. The resulting DRAAI model, constructed using logistic regression with L2 regularization, demonstrated an area under the receiver operating characteristic curve (AUROC) of 0.79. While AUROC serves as a standardized metric quantifying discriminative predictive validity between healthy and affected populations, image-based methodologies typically exhibit superior classification accuracy.

A notable example emerged from National Taiwan University Hospital, where investigators developed a convolutional neural network (CNN)-based assessment platform [14]. The study compiled 327 clinical images from hospitalized patients’ medical documentation, with three clinical specialists providing collaborative annotation for binary erythema classification and categorical necrosis assessment (extensive, moderate, or limited). The Inception-ResNet-v2 architecture achieved remarkable accuracy: 98.5% accuracy for erythema detection and 94.8% for necrotic tissue classification. Comparative analysis against AlexNet, VGG19, and ResNet50 architectures, evaluated through F1-score metrics, confirmed the superiority of the selected model. These studies exemplify the progressive enhancement of image-based AI diagnostic systems.

Object detection algorithms have particularly advanced the practical applicability of AI-driven pressure ulcer assessment. Responding to increasing clinical demands associated with demographic aging, a 2022 study implemented YOLOv4 object detection for pressure ulcer staging [15]. The integration of high-resolution smartphone cameras with mathematical modeling and computer vision algorithms has facilitated the development of sophisticated wound assessment applications. The YOLOv4-based real-time detection system demonstrated specificity exceeding 85% across all stages and sensitivity approaching 70%, establishing the viability of mobile-based diagnostic platforms.

Subsequent developments in 2023 featured a YOLOv5s-based framework targeting five categorical classifications: Stages 1–4 and unstageable lesions [16]. The study employed approximately 1000 images enhanced through data augmentation techniques, with expert clinicians providing bounding box annotations. Accuracy metrics included mean average precision (mAP) of 0.769 at IoU threshold 0.5 (mAP@0.5) and 0.398 for mAP@0.5:0.95. A more recent 2025 publication compared multiple YOLOv8 variants utilizing COCO pre-trained parameters [17]. Despite dataset limitations (720 samples), the study emphasized hyperparameter optimization with batch sizes of 16 and 150 training epochs. YOLOv8s achieved optimal performance with DTI classification accuracy of 0.90, unstageable accuracy of 0.91, and Stages 1–4 accuracies ranging from 0.70 to 0.77. These findings suggest that DTI and unstageable categories possess more distinctive visual characteristics compared to traditional staging classifications, indicating potential benefits from selective class inclusion strategies.

Concurrently, Mersey Care NHS Foundation Trust evaluated Faster R-CNN architectures integrated with mobile platforms for automated pressure ulcer classification and documentation [18]. The study acknowledged persistent knowledge gaps among healthcare professionals despite established treatment protocols. Training utilized 216 samples collected over eight months across six classification categories: Stages 1–4, DTI, and unstageable. Classification capability evaluation yielded precision of 0.6796, recall of 0.69, and F1-score of 0.67.

The collective body of research indicates substantial momentum in AI-driven pressure ulcer detection and classification, with diverse methodological approaches producing clinically relevant outcomes. Building upon these foundational studies, our study seeks to develop an optimized object detection-based classification system while validating its preliminary clinical applicability through comprehensive mobile application deployment and real-world performance assessment. In summary, while prior studies provided foundational metrics, they were limited by constrained dataset sizes and experimental settings. This detailed analysis of previous methodologies highlights the specific need for a robust, clinically validated model with higher classification accuracy, which our study addresses.

## 2. Materials and Methods

### 2.1. Preprocessing Data

This study was reviewed and approved by the Institutional Review Board of Eulji Medical Center, Daejeon (Approval No. EMC IRB-2025-06-003). Informed consent was waived due to the retrospective design of the study and the use of fully anonymized, de-identified patient data. A total of 1282 pressure ulcer images were extracted from the electronic health record systems of three geographically distinct campuses, Eulji University Hospital Daejeon in the central Chungcheong region, Eulji University Hospital Nowon in northeastern Seoul, and Eulji University Hospital Uijeongbu in northern Gyeonggi Province, in approximately equal proportions. This sampling strategy captured inter-campus variability in patient demographics and regional wound care practice patterns. All images were systematically categorized by a board-certified wound care specialist within the Quality Improvement division according to established NPUAP and EPUAP staging protocols. The dataset distribution is shown in Table 1.

As shown, Stage 2 lesions are the most prevalent (55.7%), reflecting typical hospital settings. To mitigate potential bias arising from this class imbalance, we applied data augmentation techniques, including Mosaic and Mixup, during the training phase. Deep Tissue Injury (DTI) and unstageable categories were excluded due to insufficient sample sizes, which posed a risk of overfitting [19].

Image annotation was performed using the Label Studio platform. A trained researcher positioned bounding boxes around individual lesions, with periodic validation by a lead clinician to ensure inter-rater consistency. All pressure ulcers were classified in strict accordance with the NPUAP guidelines [2].

### 2.2. Building Model

This study employed four machine learning-based object detection architectures for comparative analysis of pressure ulcer staging classification, YOLOv5x, YOLOv7, YOLOv8x, and YOLOv11x, and Faster R-CNN. YOLO frameworks have gained substantial traction in medical imaging due to their computational efficiency and classification precision. YOLOv5x offers streamlined architecture and practical implementation advantages, while YOLOv7 indicates enhanced accuracy through architectural refinements. YOLOv8x introduces a fully anchor-free architecture with decoupled classification and regression heads, enhanced label assignment, and improved gradient propagation stability. As a recent iteration of the YOLO family, it suggests superior generalization ability, faster inference, and strong performance in small dataset clinical environments, although its applications in medical imaging remain relatively limited in the literature. YOLOv11x constitutes Ultralytics’ latest advancement with anchor-free structural paradigms, though clinical applications remain underexplored Faster R-CNN serves as a benchmark two-stage detection methodology with proven predictive validity across medical imaging applications, including COVID-19 diagnosis, pulmonary nodule detection, and malaria classification [20,21,22].

The analytical framework incorporated architectural characteristics, convergence patterns, and comprehensive performance metrics including precision, recall, and mean average precision (mAP). Although deep learning frameworks typically require extensive labeled datasets, transfer learning methodologies enable superior classification accuracy with constrained sample sizes [23]. Each model was initialized with pre-trained COCO dataset parameters prior to training. To ensure a controlled and reproducible comparison, all experiments were conducted under standardized conditions, using the same data augmentation pipeline, a consistent input image resolution (640 × 640), and identical hyperparameters across all models (200 epochs, batch size 64), as detailed in Table 2. We deliberately chose not to perform model-specific hyperparameter optimization; the goal of this comparison is to evaluate the baseline out-of-the-box transfer learning performance of each architecture on our clinical dataset, thereby isolating architectural differences as the sole variable. As a result, the reported performance metrics reflect each model’s default transfer learning capability and should not be interpreted as the absolute maximum achievable accuracy for each architecture. All computational experiments utilized an NVIDIA RTX 3090 Ti GPU workstation with the Ultralytics PyTorch framework.

The model was implemented as illustrated in Algorithm 1. The dataset was split 8:2 into training and validation sets with patient-level nonoverlap and stage-wise stratification, such that all images from a single patient were assigned exclusively to either the training or the validation set. All intermediate metrics and the operating threshold were computed on the validation set only, with no post hoc tuning applied to any subsequent data. The prospectively collected clinical pilot cohort (46 cases; Section 3.2) served as a fully independent test set: these cases were collected after model development was complete, under real-world ICU deployment conditions, and were assessed by clinical nursing staff rather than researchers. No images from the pilot cohort were used at any stage of model training or validation. This three-stage separation—training set, validation set, and prospective clinical test set—structurally prevents data leakage and ensures that final performance metrics reflect genuine out-of-sample generalization. For each model, performance metrics including precision, recall, mAP@0.5, and mAP@0.5:0.95 were computed and recorded for comparison.
**Algorithm 1.** Stage Classification and Evaluation with Object Detection Models1:**function** ASSESSPRESSUREULCERSTAGES2:   dataset ← IMPORTDATA (“pressure_ulcer_images”)3:   labels ← [Stage1, Stage2, Stage3, Stage4]4:   train_data, valid_data ← PARTITION (data, split = 0.8)5:   candidates ← [YOLOv5x, YOLOv7, YOLOv8x, YOLOv11x, Faster R-CNN]6:   **for** each model in candidates do7:      TRAIN (model, train_data)8:      predictions ← PREDICT (model, valid_data)9:      precision ← COMPUTEPRECISION (predictions, valid_data.labels, labels)10:      recall ← COMPUTERECALL (predictions, valid_data.labels, labels)11:      mAP@0.5 ← COMPUTEMAP (predictions, valid_data.labels, iou = 0.5)12:      mAP@0.5:0.95 ← COMPUTEMAP (predictions, valid_data.labels, iou_range = [0.5, 0.95])13:      STORERESULTS (model.name, precision, recall, mAP@0.5, mAP@0.5:0.95)14:   **end for**15:   COMPARERESULTS16:**end function**

#### 2.2.1. YOLOv5x

YOLOv5 adopts an anchor-based architecture with a CSPDarknet backbone and PANet feature aggregation, providing stable optimization and efficient computation for pressure ulcer detection [24]. The model was trained using standard sigmoid activation and Binary Cross-Entropy loss, with class-balanced batch sampling to prevent classification capability bias toward majority classes. The final classification objective was computed as the sum of per-class losses, as shown in Equation (1).

(1) $$L_{cls}=\sum_{i=1}^{C}l_{i}$$

This formulation was consistently applied during model training to maintain uniform optimization across all classes.

#### 2.2.2. YOLOv7

YOLOv7 introduces an enhanced training strategy through its Extended Efficient Layer Aggregation Network (E-ELAN), compound scaling, and auxiliary supervision [25]. A key distinction from YOLOv5 is the use of Optimal Transport Assignment (OTA), which replaces heuristic matching with a cost-driven anchor–ground truth optimization mechanism. The matching cost includes both classification and localization components, as described in Equation (2).

(2) $$C_{ij}=\lambda_{cls}l_{cls}\left(p_{i},\;y_{j}\right)+\lambda_{iou}[1-\mathrm{IoU}(b_{i},b_{j})]$$

Optimal assignment is achieved by minimizing the total transport cost, as expressed in Equation (3).

(3) $$\underset{{X\in \{0,1\}}^{N\times M}}{\min}\sum_{i,j}C_{ij}X_{ij}$$

This assignment structure contributed to improved bounding box localization and classification stability during model training.

#### 2.2.3. YOLOv8x, YOLOv8n

YOLOv8 incorporates an anchor-free structure and a decoupled detection head, separating classification and localization gradients to improve convergence stability. To jointly optimize accuracy in class prediction, geometric alignment, and bounding box regression, a unified loss function was applied as shown in Equation (4) [26,27].

(4) $$L=L_{\mathrm{cls}}+L_{bbox}+\lambda L_{\mathrm{IoU}}$$

This combined formulation enabled adaptive balancing of detection confidence and spatial precision during training.

#### 2.2.4. YOLOv11x

The YOLOv11 model used in this study applies the Simplified Optimal Transport Assignment (SimOTA) strategy, which dynamically adjusts the number of matched anchors using aggregated IoU values [28]. The number of selected anchors per ground truth instance is described in Equation (5).

(5) $$k_{j}=\max(1,\mathrm{round}(\sum_{i=1}^{N}\mathrm{IoU}(b_{i}{,b}_{j})))$$

This adaptive matching procedure reduced computational overhead while maintaining reliable detection classification capability during training.

#### 2.2.5. FASTER R-CNN

Faster R-CNN follows a two-stage detection process, where the Region Proposal Network generates candidate bounding boxes, followed by refined classification and localization in the second stage [29]. The final learning objective combines a classification term and a bounding box regression term, weighted by the balancing factor λ, as shown in Equation (6).

(6) $$L\left(\left\{p_{i}\right\},\left\{t_{i}\right\}\right)=\frac{1}{N_{cls}}\sum_{i}L_{cls}\left(p_{i},p_{i}^{*}\right)+\lambda \frac{1}{N_{reg}}\sum_{i}p_{i}^{*}L_{reg}(t_{i},t_{i}^{*})$$

This multi-task formulation enabled high localization precision while preserving classification reliability.

## 3. Results

### 3.1. Model Result

**Figure 1:**
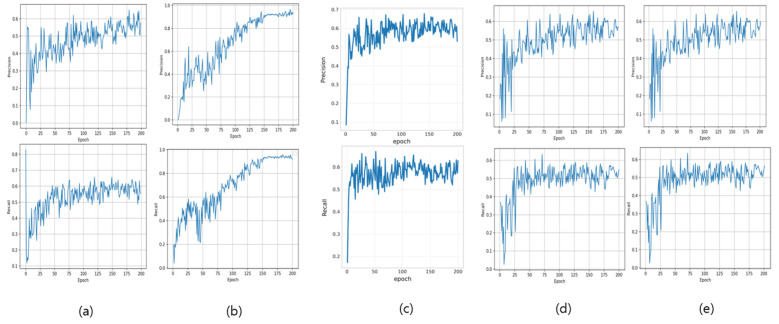
Precision and recall per epoch. (**a**) shows the results of YOLOv5x, (**b**) YOLOv7, (**c**) YOLOv8x, (**d**) YOLOv11x, and (**e**) Faster R-CNN. It can be observed that the values converge as training progresses. For YOLOv7, mAP@0.5 increases from ~0.75 to 0.96 by epoch 80 and then changes by <0.01 thereafter, indicating convergence; its final mAP@0.5:0.95 ≈ 0.68 exceeds other models, hence the best overall accuracy.

Figure 1 illustrates the precision and recall accuracy trajectories over the training epochs for the five evaluated artificial intelligence architectures: YOLOv5x, YOLOv7, YOLOv8x, YOLOv11x, and Faster R-CNN. Precision quantifies the ratio of correctly identified positive instances relative to the total number of positive predictions generated by the model. Recall constitutes the fraction of actual positive cases that were successfully detected and classified by the algorithmic framework.

**Figure 2:**
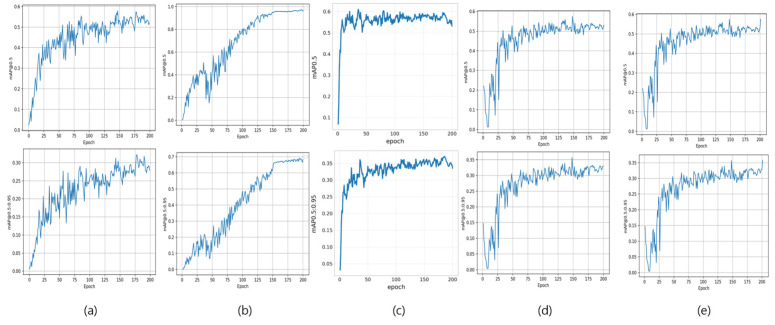
mAP@0.5 and mAP@0.5:0.95 per Epoch. (**a**) shows the results of YOLOv5x, (**b**) YOLOv7, (**c**) YOLOv8x, (**d**) YOLOv11x, and (**e**) Faster R-CNN.

Figure 2 depicts the mAP@0.5 and mAP@0.5:0.95 predictive validity evolution throughout the training progression for the identical four architectural frameworks. The mAP@0.5 metric reflects the arithmetic mean of class-specific average precision values, computed utilizing an Intersection over Union (IoU) threshold criterion of 0.5. IoU serves as a quantitative measure assessing the spatial correspondence between algorithmically predicted bounding box coordinates and ground truth annotation boundaries. The mAP@0.5:0.95 metric constitutes the averaged mAP calculations across IoU threshold values spanning from 0.5 to 0.95 at 0.05 incremental intervals, thereby establishing a comprehensive and stringent performance assessment framework.

The visualization reveals distinct convergence patterns across the evaluated architecture. Models (a), (c), and (d) demonstrate comparable performance trajectories, with mAP@0.5 values stabilizing within the 0.5–0.6 range and mAP@0.5:0.95 metrics converging between 0.30–0.35. In marked contrast, architecture (b) exhibits substantially superior predictive validity characteristics, achieving mAP@0.5 convergence approaching 0.96 and mAP@0.5:0.95 values reaching approximately 0.68, indicating exceptional classification capability and robust generalization across varying IoU threshold criteria.

**Figure 3:**
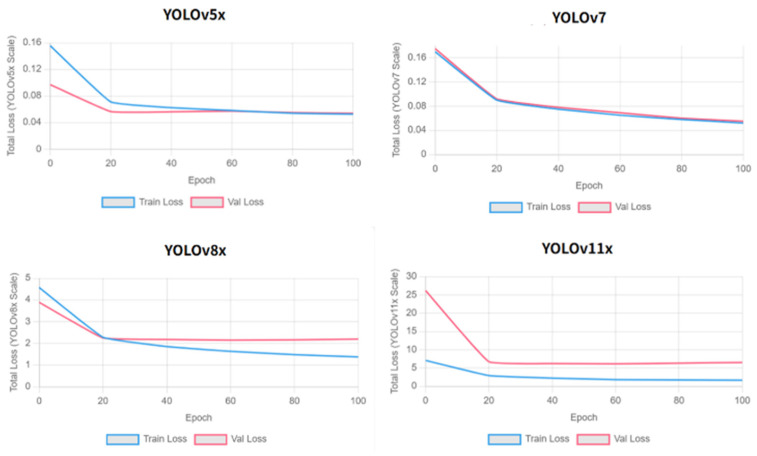
Training and validation loss curves for YOLOv5x, YOLOv7, YOLOv8x, and YOLOv11x. YOLOv7 shows the most stable learning behavior with minimal gap between curves, while YOLOv5x and YOLOv11x exhibit divergence indicative of overfitting. YOLOv8x shows intermediate stability.

To investigate the mechanistic reason for this significant classification capability gap, we further analyzed the learning dynamics of the YOLO-series models (Figure 3). The loss curves provide a clear explanation: both YOLOv5x (a) and YOLOv11x (c) exhibit clear signs of early overfitting, where the validation loss (red line) begins to stagnate or rise after 40–60 epochs, while the training loss (blue line) continues to decrease. In stark contrast, YOLOv7 (b) shows stable convergence, with both training and validation losses decreasing together without signs of overfitting. This analysis strongly suggests that the superior mAP of YOLOv7 (0.96) is attributable to its architectural ability to generalize better to our dataset, whereas the other models suffered from overfitting, limiting their performance.

According to Table 3, YOLOv5x, YOLOv8x, YOLOv11x, and Faster R-CNN exhibited mAP@0.5 convergence within 0.5 to 0.6, with mAP@0.5:0.95 values between 0.30 and 0.35. In contrast, YOLOv7 achieved mAP@0.5 of 0.97 and mAP@0.5:0.95 of 0.68. As each model was trained once under standardized conditions without repeated runs, confidence intervals for these metrics could not be computed. The robustness of this accuracy difference across multiple training runs should be verified in future work. Detailed accuracy analysis across individual pressure ulcer stages was conducted using confusion matrix evaluation.

**Figure 4:**
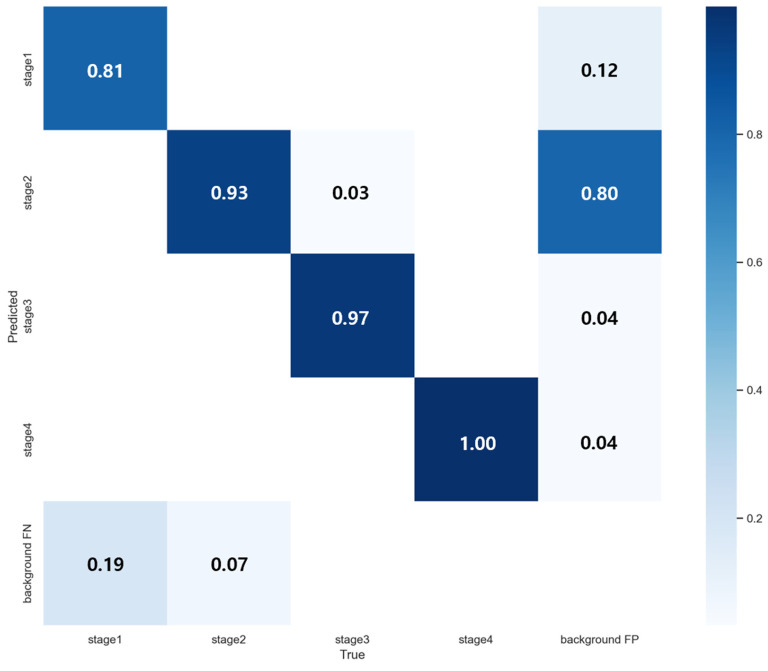
YOLOv7 Confusion Matrix.

**Figure 5:**
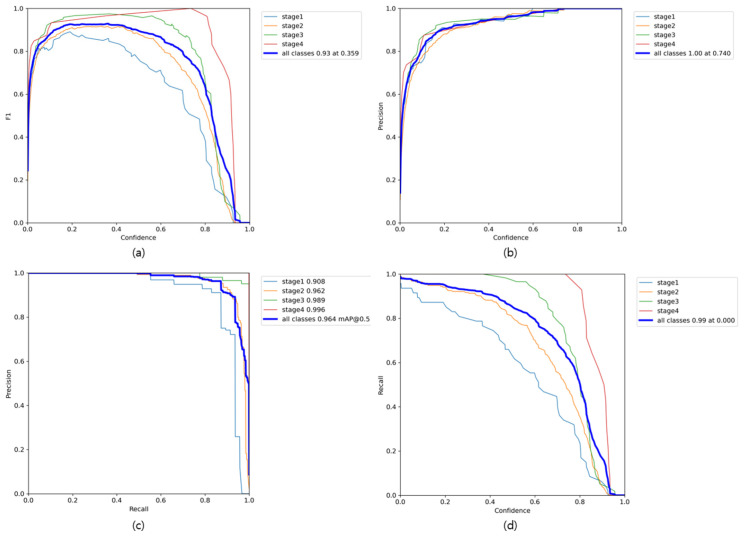
Analysis of Class-wise Precision, Recall, F1-score, and mAP. (**a**) shows the F1-score across varying confidence thresholds. (**b**) presents the precision values according to different confidence thresholds. (**c**) illustrates the precision–recall curve, including the class-wise AP@0.5 values. (**d**) depicts the recall performance across confidence thresholds.

As shown in Figure 4, the confusion matrix reveals exceptional classification accuracy across all staging categories, with particularly strong classification capability in Stage 2 identification, the most diagnostic challenging category for nursing personnel. YOLOv7 achieved 93% accuracy for Stage 2 classification, demonstrating capability in distinguishing subtle morphological characteristics that typically confound visual assessment. Stage 4 lesions yielded perfect 100% classification accuracy due to their distinct visual features. Comprehensive performance metrics are presented in Figure 5.

Figure 5 presents a comprehensive class-wise performance evaluation encompassing precision, recall, F1-score, and mean average precision (mAP) metrics. The F1-score analysis depicted in Figure 4a shows sustained high-performance levels across all classification categories, achieving optimal performance of 0.93 at a confidence threshold of 0.359. Figure 5b,d reveal that both precision and recall metrics maintain elevated values even when confidence thresholds exceed 0.8, indicating robust model reliability under stringent classification criteria. Notably, overall class precision achieves perfect performance (1.00) at a threshold of 0.740, demonstrating the model’s capacity for stable and dependable predictions at elevated confidence levels. The precision-recall relationship illustrated in Figure 5c exhibits exceptional balance. The overall mean average precision (mAP@0.5) achieved an outstanding 0.964. As requested by the reviewer, we analyzed the class-specific performance, which was already included in the legend of Figure 5c. The class-wise AP@0.5 values were 0.908 for Stage 1, 0.962 for Stage 2, 0.989 for Stage 3, and 0.996 for Stage 4. This detailed analysis confirms that the model’s superiority is not due to any single class, but shows robust and high accuracy across all stages, including the most diagnostically challenging Stage 2.

We performed a clinically oriented error analysis by decomposing false negatives for Stage 2 and Stage 3 into three types: missed detections, adjacent-stage misclassifications between Stage 2 and Stage 3, and non-adjacent stage misclassifications. One-to-one matching was applied at an IoU threshold of 0.5, and predictions below a confidence threshold of 0.25 were excluded, with the resulting breakdown summarized in Table 4. For Stage 2, missed detections accounted for the majority of false negatives in YOLOv5, YOLOv8n, and YOLOv11, indicating that their limitations were driven primarily by failures to detect the lesion rather than by uncertainty at the Stage 2 versus Stage 3 boundary. In contrast, YOLOv7 substantially reduced missed detections while maintaining a low rate of adjacent-stage misclassification. For Stage 3, we report under-staging separately, defined as Stage 3 cases predicted as Stage 2, because such errors may delay escalation in clinical workflows; YOLOv7 exhibited the lowest under-staging frequency among the evaluated models. Overall, Table 1 indicates that performance differences across models reflect not only aggregate detection accuracy but also the distribution of missed detections and stage-confusion errors, with the Stage 2 to Stage 3 boundary representing a key source of clinically relevant misclassification.

**Figure 6:**
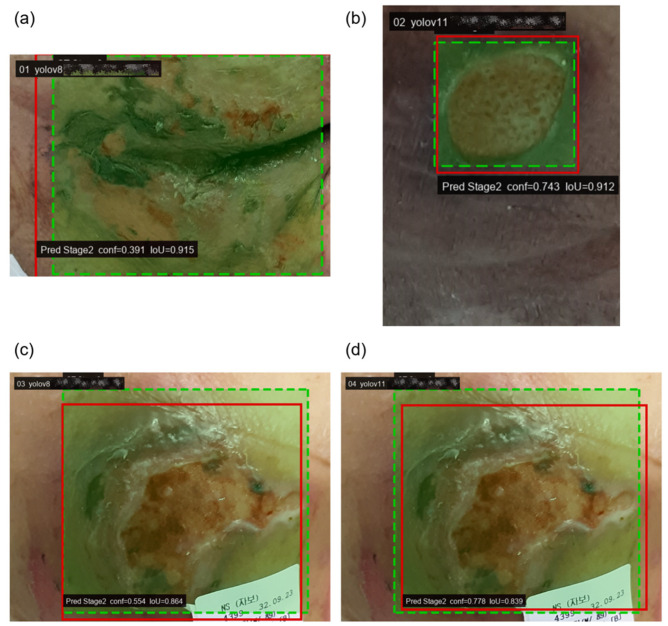
Qualitative examples of under-staging errors (Stage 3 misclassified as Stage 2) with high localization overlap.

**Figure 7:**
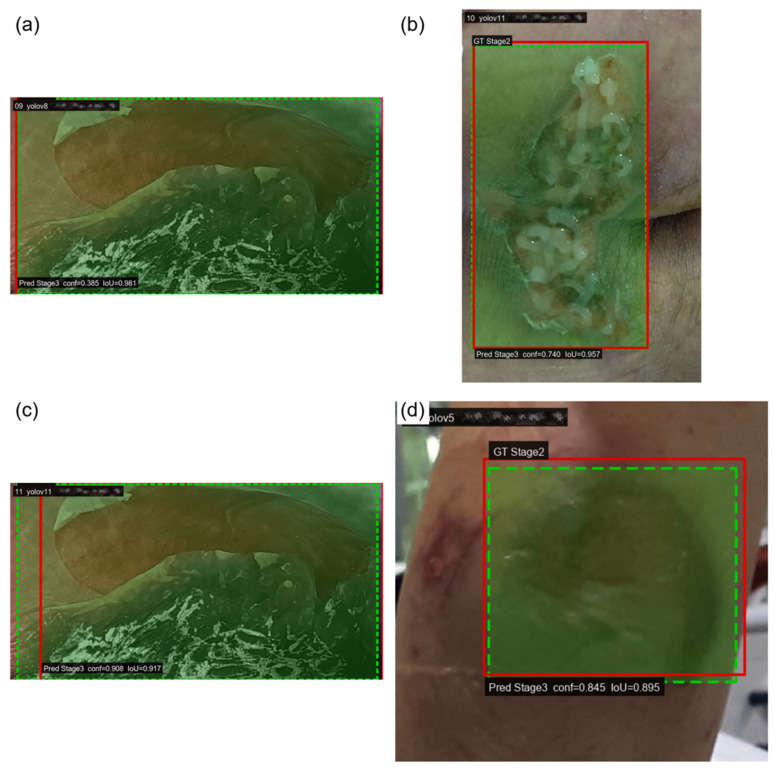
Qualitative examples of over-staging errors (Stage 2 misclassified as Stage 3) under high IoU matching.

Table 4 indicates that a substantial portion of errors for Stage 2 and Stage 3 arises from two distinct mechanisms: missed detections and adjacent-stage misclassifications between Stage 2 and Stage 3. Because these two stages constitute a clinically important boundary and are frequently challenging to differentiate by visual appearance alone, we further examined representative failure cases to clarify whether errors were driven primarily by localization failure or by ambiguity in stage-level visual cues. Figure 6 and Figure 7 present qualitative examples selected from high-overlap matches, in which the predicted and ground truth bounding boxes largely coincide while the predicted class shifts to the adjacent stage. These examples illustrate that certain failures persist even when lesion localization is accurate, supporting the interpretation that misclassification at the Stage 2 versus Stage 3 boundary can be dominated by fine-grained visual ambiguity rather than detection error. For readability, the predicted bounding boxes are slightly dilated in the figures for visualization only, and this adjustment is not used in any quantitative evaluation.

Green dashed boxes indicate ground truth annotations and red solid boxes indicate model predictions. Despite high IoU values, the predicted stage is shifted to an adjacent category, suggesting that these failures are primarily driven by stage boundary ambiguity rather than localization errors. Panels (a) and (c) show predictions from YOLOv8, while panels (b) and (d) show predictions from YOLOv11. For visualization clarity, the predicted bounding boxes are slightly dilated only in the figure.

Green dashed boxes represent ground truth annotations and red solid boxes represent predictions. The overlap between boxes remains high, yet the classification shifts to the adjacent stage, highlighting that a substantial portion of errors arises from discriminating Stage 2 versus Stage 3 appearance rather than detecting the lesion region. Panels (a) and (c) show predictions from YOLOv8, panel (b) shows a prediction from YOLOv11, and panel (d) shows a prediction from YOLOv5. Predicted boxes are slightly dilated only for visualization.

**Figure 8:**
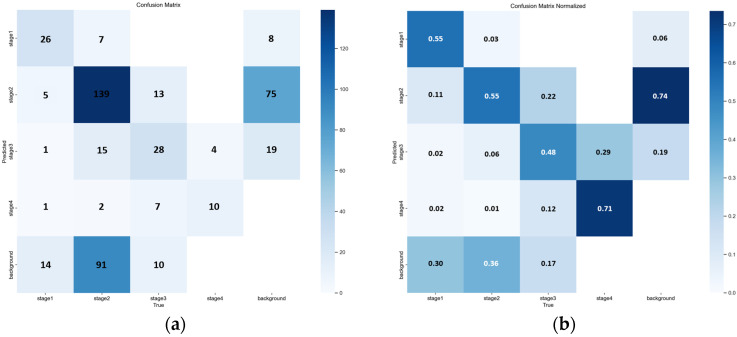
Confusion matrix of YOLOv8n for pressure ulcer stage classification. (**a**) Confusion matrix of YOLOv8n for pressure ulcer stage classification (**b**) Confusion matrix normalized.

**Figure 9:**
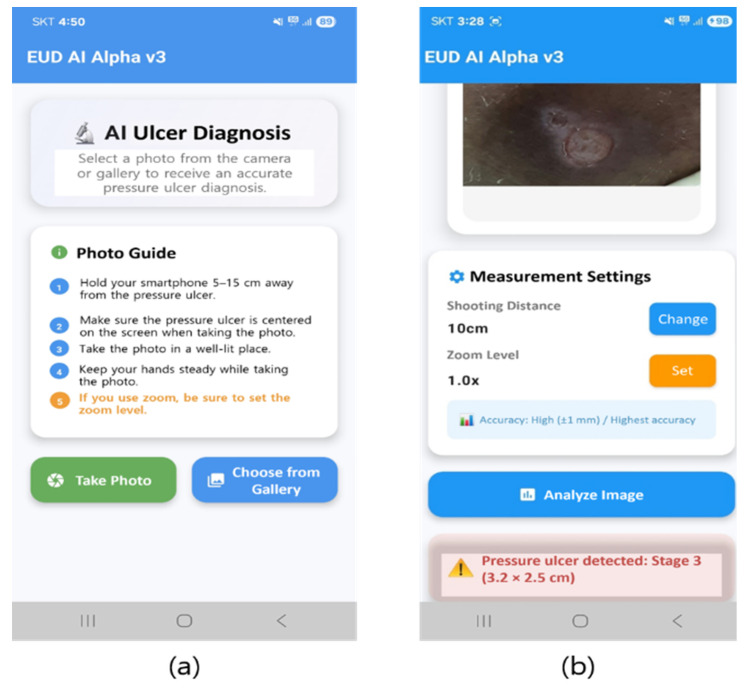
Mobile Application Interface and Clinical Implementation. (**a**) Subfigure a presents the main interface of the mobile application, including AI-based diagnostic guidance and image capture instructions. (**b**) Subfigure b shows the real-time analysis screen displaying automated stage classification and wound size measurement results.

The matrix highlights frequent misclassification between Stage 2 and Stage 3, consistent with the stage boundary errors discussed in Table 4 and illustrated in Figure 6 and Figure 7. A normalized version is provided to facilitate comparison of per-class confusion patterns. The quantitative breakdown in Table 4 and the qualitative examples in Figure 6 and Figure 7 collectively indicate that a non-trivial fraction of errors is concentrated at the Stage 2 versus Stage 3 boundary, including clinically relevant under-staging. Consistent with this observation, the YOLOv8n confusion matrices in Figure 8 show frequent cross-stage confusion between Stage 2 and Stage 3, while the F1-confidence curve in Figure 9 suggests that overall performance is sensitive to the selected confidence threshold and exhibits an operating point near a moderate threshold range. Although YOLOv8n offers clear efficiency advantages, these results support the conclusion that reliable bedside deployment for stage-level clinical decision support is likely to require a higher-performing model, together with a hardware environment capable of sustaining real-time inference without compromising diagnostic reliability. This motivates the following comparison with prior studies and the subsequent clinical pilot validation.

Comparison with State-of-the-Art: As detailed in Table 5, our study compares the proposed framework with representative object detection studies. While recent research utilizing YOLOv5 and YOLOv8 architectures typically reported mAP@0.5:0.95 values ranging from 0.5 to 0.7 [16,17], our YOLOv7-based model demonstrated superior classification accuracy with an mAP@0.5 of 0.96 and mAP@0.5:0.95 of 0.68. This significant performance margin validates the effectiveness of our optimized training strategy compared to existing baselines.

Clinical Implementation & Feedback: Following the model evaluation, we deployed the mobile application at Eulji University Daejeon Medical Center to validate its real-world utility. Clinical staff successfully integrated the app into their routine assessments, providing positive feedback on its role as an intuitive decision support tool. Crucially, healthcare professionals noted that the AI provided meaningful assistance in differentiating Stage 2 and Stage 3 lesions, a frequent source of diagnostic ambiguity in visual assessment. During this pilot deployment, the application maintained a consistent diagnostic accuracy of 87%, demonstrating its practical value in a live clinical environment.

### 3.2. Clinical Pilot Study and Mobile Application Validation

To evaluate the initial feasibility and usability of the YOLOv7-based pressure ulcer classification system in a real clinical environment, a prospective feasibility pilot study was conducted in the Intensive Care Unit (ICU) of Eulji University Daejeon Medical Center. This pilot was designed as a first-in-practice deployment assessment, consistent with standard methodology for initial clinical AI validation studies, rather than a definitive large-scale clinical trial. The application was designed to integrate into existing workflows, providing intuitive AI-powered diagnostic support.

The study spanned two weeks following Institutional Review Board approval. Ten registered nurses with varying levels of experience voluntarily participated, providing informed consent and receiving standardized training on the application and image capture protocols. During routine wound assessment rounds, nurses first performed a standard visual assessment and independently recorded their staging diagnosis prior to obtaining the AI classification. This sequential protocol was designed to capture both nurse-alone and AI-alone performance against the same expert ground truth. However, due to the small pilot sample size (46 cases), a formal statistical comparison of nurse-alone versus AI-alone accuracy was underpowered and is reported descriptively. Future studies should be powered to formally compare nurse-alone accuracy, AI-alone accuracy, and nurse-with-AI accuracy against the same expert ground truth to fully characterize the additive clinical value of AI assistance. A total of 46 distinct pressure ulcer cases were collected. To ensure validation accuracy, a board-certified wound care specialist (WCS) served as the sole validator for the Ground Truth (GT), adhering to the protocol established in our preliminary analysis. The specialist reviewed all 46 images while blinded to both the AI predictions and the nurses’ initial assessments. This single-expert, blind protocol provided a consistent reference standard. The primary endpoint was the diagnostic accuracy of the AI model relative to the GT. Secondary endpoints included the accuracy of the nurses’ assessments, user satisfaction (5-point Likert scale), and workflow efficiency. Accuracy was defined as the proportion of correct classifications relative to the GT.

Compared to the expert-defined GT, the AI assessment achieved an overall diagnostic accuracy of 87.0% (40/46; 95% CI: 73.7% to 95.1%) with zero critical misclassifications, where critical misclassification is defined as under-staging by two or more stages. The average image processing time, measured from image capture initiation to AI classification output, was 3 s. User satisfaction surveys yielded a mean score of 4.0/5. Nursing staff reported that the AI-assisted workflow reduced overall assessment and documentation time from 4 to 6 min to approximately 1 min; this estimate was obtained through structured self-report by participating nurses at the end of each assessment round, comparing their perceived time for AI-assisted versus their recalled time for traditional assessment. A formal time-motion study with objective component-level measurement, including patient positioning, image capture, AI processing wait time, and documentation, was not conducted, and this time estimate should therefore be interpreted as indicative rather than definitive. Cases in which AI confidence was low and specialist review was sought were not separately timed and are not reflected in this estimate.

Figure 9 shows the mobile application interface developed for clinical pressure ulcer staging assessment. (a) The main interface features AI-powered diagnosis capabilities with user guidelines for optimal image capture, including smartphone positioning (5–15 cm distance), proper lighting conditions, and optimal angles. (b) Real-time analysis interface displays captured images with measurement settings and automated AI classification results. The system accurately identified a Stage 3 pressure ulcer with precise dimensional measurements (3.2 × 2.5 cm), demonstrating capabilities for both staging classification and wound size quantification.

A systematic clinical validation study was conducted over 2 weeks, involving 10 registered nurses with varying experience levels. The application processed 46 real-world pressure ulcer cases, achieving an average processing time of 3 s per image and 87% clinical accuracy agreement with expert assessment. User satisfaction surveys revealed a 4.0/5 rating, and the AI-assisted assessment reduced traditional staging time from 4–6 min to approximately 1 min.

Post-deployment evaluation revealed the application’s dual value as a diagnostic support tool and educational resource. Nursing staff emphasized its effectiveness in supporting novice nurses with pressure ulcer stage differentiation. The system provided meaningful guidance in ambiguous cases, enhanced clinical confidence, and improved assessment protocol standardization. Zero critical misclassifications were recorded during deployment, confirming the system’s safety and successful integration into clinical workflows.

## 4. Discussion

This investigation constitutes a comprehensive evaluation of object detection architectures for pressure ulcer staging classification, culminating in the development and clinical validation of a practical mobile application. The findings demonstrate that artificial intelligence-driven assessment tools can achieve high diagnostic accuracy while simultaneously addressing critical educational and workflow challenges within contemporary healthcare environments.

The superior performance of the YOLOv7 architecture (mAP@0.5: 0.96, mAP@0.5:0.95: 0.68) compared to alternative frameworks establishes its suitability for clinical implementation. Particularly significant is the model’s high accuracy in Stage 2 classification (93%), which reflects the most diagnostic challenging category for nursing personnel. This finding highlights the model’s potential as a clinical decision support tool, enhancing diagnostic reliability and reducing uncertainty, particularly in ambiguous Stage 2 cases. The ability to consistently differentiate these lesions shows the model’s capacity to mitigate misclassification and guide appropriate treatment protocols.

The mobile application was deployed at Eulji University Daejeon Medical Center. Mobile application-based approaches for pressure injury management have previously demonstrated feasibility in clinical settings, supporting the translational potential of the current deployment. This deployment provides robust evidence of the system’s real-world applicability and preliminary clinical applicability. The systematic reduction in assessment time from 4–6 min to approximately 1 min serves as a significant improvement in clinical efficiency, particularly valuable in intensive care environments. Furthermore, the high user satisfaction rating (4.0/5) and successful workflow integration confirm that AI-assisted diagnostic tools can be seamlessly incorporated into existing clinical protocols without disrupting patient care.

Of clinical significance is the application’s value as an educational resource for novice nursing personnel. The consistent provision of immediate feedback and diagnostic guidance addresses a recognized gap in pressure ulcer education. This extends the system’s utility beyond simple diagnostic support, positioning it as a comprehensive training platform that can improve overall nursing competency and standardize the decision-making criteria for pressure ulcer staging among new nurses. The zero critical misclassifications recorded during deployment further support the system’s safety profile for clinical implementation.

The comparative analysis with existing literature reveals that our YOLOv7-based approach achieves superior performance metrics. While recent investigations utilizing YOLOv5s and YOLOv8 variants have reported mAP@0.5 values ranging from 0.77 to 0.84, our implementation achieved 0.96, representing a significant advancement in classification accuracy. This improvement likely stems from the combination of optimized architecture selection, comprehensive dataset curation, and systematic hyperparameter optimization.

Despite these promising findings, several methodological limitations warrant consideration. A key limitation, as the reviewer correctly pointed out, is the utilization of images from a single healthcare institution. This inherently limits the generalizability of our findings, as imaging conditions, patient demographics, and skin pigmentation may vary across different institutions. Ideally, validation on an external, multi-center dataset would be required to definitively confirm the model’s robustness. However, due to strict patient privacy regulations and institutional data sharing policies, acquiring a suitable external dataset was not feasible within the timeframe of this study. We have thus clearly stated this as a primary limitation and strongly suggest multi-institutional data collection as a critical direction for future research. Furthermore, adopting advanced data augmentation techniques, such as saliency-guided approaches [30], could be explored to enhance model generalization across diverse clinical settings.

The current framework’s focus exclusively on pressure ulcer pathology represents another limitation. Clinical practice frequently requires differential diagnosis with alternative dermatological conditions, and integrating multi-pathology recognition would significantly enhance the system’s preliminary clinical applicability. Additionally, the exclusion of Deep Tissue Injury (DTI) and unstageable categories due to insufficient sample sizes reflects a gap that should be addressed in future research. It is important to acknowledge that this study evaluated established object detection architectures without proposing novel architectural innovations. The contribution of this work lies in systematic comparative evaluation of contemporary models and rigorous clinical translation of the best-performing architecture to real-world nursing workflows. While we demonstrate that YOLOv7 achieves a superior performance among the evaluated models for pressure ulcer classification, this finding should be interpreted within the scope of our comparative analysis rather than as a claim of absolute optimality across all possible architectures. Future work may explore architecture modifications specifically optimized for medical imaging characteristics, including pressure ulcer-specific feature extraction mechanisms or multi-scale attention modules tailored to wound assessment.

The clinical deployment study, while demonstrating positive outcomes, was conducted over a relatively brief period (2 weeks) with a limited number of participants (10 nurses). Extended longitudinal studies involving larger cohorts across multiple clinical units are needed to provide more robust evidence of long-term effectiveness and sustainability. Furthermore, an economic analysis comparing the cost-effectiveness of AI-assisted assessment versus traditional protocols would strengthen the argument for widespread clinical adoption.

Technical architecture suggests promising scalability, with real-time processing capabilities (3 s per image) suitable for clinical environments. However, the current implementation requires standard smartphone devices, which may limit accessibility in resource-constrained settings. Future development should consider optimization for lower-specification devices and integration with existing hospital information systems to maximize accessibility.

The educational implications of this research extend beyond immediate clinical applications. The effectiveness of AI-assisted training for novice nurses suggests potential applications in formal novice nurse training support and continuing professional development. The standardization of assessment protocols facilitated by AI guidance could significantly improve inter-observer reliability and reduce practice variability across healthcare institutions.

Looking forward, several research directions emerge. The integration of advanced imaging modalities, such as infrared thermography or hyperspectral imaging, could enhance diagnostic accuracy and provide additional wound characterization capabilities. The development of predictive models that estimate healing trajectories based on initial staging and patient characteristics serves as another promising avenue for clinical advancement.

In conclusion, this study provides preliminary multi-campus evidence that a YOLOv7-based pressure ulcer classification system can achieve high diagnostic accuracy and demonstrate feasibility in a real-world ICU environment, supporting its potential utility as a clinical decision support and nursing education tool pending external validation at independent institutions. The successful mobile application deployment confirms the feasibility of AI-assisted assessment tools and highlights their potential to address critical challenges in educational scaffolding and clinical practice. These findings establish a foundation for future research aimed at expanding the scope and accessibility of AI-driven diagnostic support systems in healthcare.

## 5. Conclusions

This study developed and conducted a prospective clinical pilot validation of an AI-based pressure ulcer staging classification system using object detection architectures, with model development data collected across three geographically distinct campuses of Eulji University Medical Center. Among the five evaluated architectures (YOLOv5x, YOLOv7, YOLOv8x, YOLOv11x, and Faster R-CNN), YOLOv7 demonstrated the strongest performance under identical training configurations applied consistently across all models, achieving an mAP@0.5 of 0.964 and mAP@0.5:0.95 of 0.68, with 93% accuracy for the diagnostically challenging Stage 2 category in internal validation.

The prospective feasibility pilot at Eulji University Hospital Daejeon, involving 10 nurses over 2 weeks and processing 46 consecutive ICU cases, yielded 87% diagnostic accuracy against expert ground truth (95% CI: 73.7% to 95.1%), 4.0/5 user satisfaction, and a reported reduction in assessment time from 4 to 6 min to approximately 1 min, with zero critical misclassifications recorded.

These findings provide preliminary evidence for the potential utility of AI-assisted pressure ulcer assessment as a clinical decision support and nursing education tool within a multi-campus Korean tertiary care setting. Primary limitations include data collection within a single health system, single-expert ground truth annotation, exclusion of DTI and unstageable categories, and the small-scale nature of the clinical pilot. Future research should prioritize external validation at independent institutions, multi-expert annotation with inter-rater reliability assessment, and expansion of the classification scope to include DTI and unstageable categories through a hierarchical two-stage detection framework.

## Figures and Tables

**Table 1:** Dataset by stage.

Stage	Count	Total Percentage
Stage1	151	11.8%
Stage2	714	55.7%
Stage3	319	24.9%
Stage4	98	7.6%

**Table 2:** Hyperparameters and Learning Rate Adjustments.

Hyperparameter	Value
Augmentation Parameters	Degrees, Translate, Scale, Fliplr, Mosaic, Mixup, Hue, Saturation, Value
Lr0	0.01
Lrf	0.1
Epoch	200
Batch Size	64
Computer environment	NVIDIA RTX 3090Ti

**Table 3:** Performance metrics by models.

	mAP@0.5	mAP@ 0.5:0.95	F1-Score	Precision	Recall	Params(M)
YOLOv5x	0.58	0.33	0.59	0.55	0.62	86.7
YOLOv7	0.97	0.69	0.94	0.96	0.92	36.9
YOLOv8x	0.53	0.34	0.57	0.53	0.63	68.2
YOLOv8n	0.52	0.29	0.52	0.53	0.50	3.2
YOLOv11x	0.56	0.34	0.55	0.56	0.55	56.9
Faster R-CNN	0.58	0.36	0.58	0.6	0.56	41.8

**Table 4:** Class-Specific Error Analysis for Stage 2 and Stage 3 Across Object Detection Models.

Model	S2 GT	S2 TP	S2 FN Miss	S2 FN 2→3	S2 FN Other	S2 Recall (%)	S3 GT	S3 TP	S3 FN Miss	S3 FN 3→2	S3 FN Other	S3 Recall (%)
YOLOv5x	254	99	133	15	7	38.98	58	28	15	6	9	48.28
YOLOv7	254	230	23	1	0	90.55	58	57	0	1	0	98.28
YOLOv8x	254	130	110	14	0	51.18	58	31	13	8	6	53.45
YOLOv11x	254	116	122	14	2	45.67	58	22	17	11	8	37.93

**Table 5:** Performance of Case Study Models and the Proposed Model for Pressure Ulcer Stage Classification.

Study	Based Model	Images	Classes	mAP@0.5	mAP@0.5:0.95	F1-score	Precision	Recall
[16]	YOLOv5s	1000+	5	0.77	0.398	0.73	0.78	0.68
[17]	YOLOv8m	2800	6	0.84	N/A	0.84	0.85	0.82
[18]	Faster R-CNN	216	6	N/A	N/A	0.67	0.67	0.69
**Our Model**	YOLOv7	1282	4	0.96	0.68	0.93	0.98	0.98

## Data Availability

The datasets used and/or analysed during the current study are available from the corresponding author on reasonable request.
